# Patient‐reported outcomes of topical therapies in actinic keratosis: A systematic review

**DOI:** 10.1111/dth.14833

**Published:** 2021-02-21

**Authors:** Ayman Grada, Steven R. Feldman, Nicola Luigi Bragazzi, Giovanni Damiani

**Affiliations:** ^1^ R&D and Medical Affairs, Almirall (US) Exton PA USA; ^2^ Department of Dermatology Wake Forest School of Medicine Winston‐Salem NC USA; ^3^ Department of Mathematics and Statistics Laboratory for Industrial and Applied Mathematics (LIAM), York University Toronto Ontario Canada; ^4^ Clinical Dermatology IRCCS Istituto Ortopedico Galeazzi Milan Italy; ^5^ Department of Biomedical, Surgical, and Dental Sciences University of Milan Milan Italy; ^6^ Department of Pharmaceutical and Pharmacological Sciences University of Padua Padua Italy

**Keywords:** actinic keratosis, adherence, compliance, patient‐reported outcomes, topical, safety, systematic review, tolerability

## Abstract

Patients' perspectives on actinic keratosis treatments may have an impact on treatment adherence and, therefore, therapeutic outcomes. We performed a systematic review to assess patients' perspectives of topical, field‐directed treatments for actinic keratoses. A literature search was conducted, and 14 studies were identified encompassing 4433 patients. Only four studies were focused on face and/or scalp, which are the locations that typically impact patients' quality of life. Four studies were clinical trials. One study utilized a validated patient‐reported outcomes (PRO) instrument specifically developed for actinic keratosis. In general, treatment adherence and patient satisfaction were better with shorter‐duration treatment regimens such as ingenol mebutate gel. Imiquimod improved quality of life in one study but not in another. No data was available on topical piroxicam. The findings underscore the need for effective and well‐tolerated, short‐duration topical treatment for actinic keratosis.

## 1 INTRODUCTION

Actinic keratoses (AKs), also known as solar or senile keratoses, are chronic, recurrent cutaneous lesions resulting from the proliferation of atypical epidermal keratinocytes due to prolonged intermittent sun exposure and may progress to cutaneous squamous cell carcinoma. AK is one of the most common reasons for dermatology office visits, especially among the elderly.^1^ AK treatments can also be quite distressing to many patients due to impact on daily activities such as work and social engagements, especially for lesions in the face and scalp.

Currently, there are two major categories of AK treatments: Lesion‐directed and field‐directed therapies.^1^ The latter include photodynamic therapy (PDT) and topical treatments such as 5‐fluorouracil, imiquimod, ingenol mebutate, diclofenac, and piroxicam; they are typically used for treating areas with multiple AKs or clinical evidence of field cancerization. Fluorouracil acts by inhibiting thymidylate synthetase limiting DNA synthesis and causing cell death.^2^, ^3^, ^4^ Imiquimod is an immune response modifier which activates Toll‐like receptor 7 (TLR‐7), causing the release of cytokines by epidermal and dermal dendritic cells; imiquimod also modulates the response of natural killer cells (NKs) and B‐lymphocytes. Ingenol mebutate disrupts plasma membranes and mitochondria and induces neutrophil‐mediated cellular cytotoxicity.^2^, ^3^, ^4^ Both piroxicam and diclofenac inhibit cyclooxygenase enzymes (COX‐1 and COX‐2). The use of Ingenol mebutate for the field treatment of actinic keratosis has been associated with increased risk of nonmelanoma skin cancer (NMSC) and has been withdrawn from the market.

Understanding patients' perspective regarding topical AK treatments may help optimize patients' adherence and clinical outcomes.^5^, ^6^, ^7^, ^8^, ^9^, ^10^, ^11^, ^12^, ^13^, ^14^, ^15^, ^16^, ^17^, ^18^, ^19^, ^20^, ^21^, ^22^, ^23^, ^24^, ^25^, ^26^ A patient‐reported outcome (PRO) is a report coming directly from the patient, concerning her health status and experience with a particular treatment. Patient‐reported outcome measures (PROMs) are instruments to assess PROs from a quantitative standpoint. To better understand patients' perspectives of AK treatment, we performed a systematic review of PROs of topical field‐directed AK treatment.

## 2 MATERIALS AND METHODS

### 2.1 Systematic review development and protocol

The present systematic review was devised based on the “Preferred Reporting Items for Systematic Reviews and Meta‐analyses” (PRISMA) guidelines and the Cochrane recommendations for developing a systematic review of patient‐reported outcome measure (PROM) studies. The systematic review was further based on a protocol, developed according to the “Preferred Reporting Items for Systematic Reviews and Meta‐analyses—Protocol” (PRISMA‐P). The protocol has been submitted to the “International Prospective Register of Systematic Reviews” (PROSPERO) for registration and is also available upon request to the Corresponding Author.

### 2.2 Inclusion and exclusion criteria

Inclusion criteria were the following: P (patients suffering from AK), I (intervention, any topical field treatment for AK), C (all the articles reporting topical therapy for AK, independently from the comparison with another drug or placebo—including imiquimod, ingenol mebutate, diclofenac, piroxicam, 5FU), O (outcomes, PROs/PROMs), and S (study design, any study design). Expert opinions, comments/commentaries, letters to editor, editorials, case reports, case series, and reviews were excluded.

### 2.3 Search strategy

Search string consisted of relevant keywords (such as “actinic keratosis” and synonyms—actinic or senile keratosis—and safety, tolerability, quality of life, patient satisfaction, patient‐reported outcome—PRO—or patient‐reported outcome measure—PROM—patient perspective, patient preference or patient perception), connected by means of appropriate Boolean operators. PubMed/MEDLINE was queried from 1 January 2010 to 6 December 2020, utilizing the “Best Algorithm” option; medical subject headings (MeSH) terms and truncated words/wild‐card option were used when appropriate (Table 1).

**TABLE 1:** Search strategy adopted in present systematic review

Search strategy item	Search strategy details
String of keywords	(“actinic keratosis” OR “solar keratosis” OR “senile keratosis”) AND (safety OR tolerability OR satisfaction OR “quality of life” OR “patient satisfaction” OR “patient‐reported outcome” OR PRO OR “patient‐reported outcome measure” OR PROM OR “patient perspective” OR “patient preference” OR “patient perception”)
Database searched	PubMed/MEDLINE
Time filter	None applied (from inception)
Language filter	None applied (any language)
Inclusion criteria	P (patients suffering from AK) I (intervention, medical field treatment for AK, such as imiquimod, diclofenac, ingenol mebutate) C (comparison) *all the articles reporting medical therapy for AK, independently from the comparison with another drug or placebo* O (outcomes, including PROs/PROMs) S (study design, any study design) Publication type: original study
Exclusion criteria	P (patients not suffering from AK) I (intervention, nonmedical treatment for AK, such as surgery, cryo‐therapy or photodynamic therapy) C (comparison, with nonmedical treatment for AK) O (outcomes other than PROs/PROMs, such as clinical outcomes) S (study design) Publication type (expert opinions, comments, commentaries, letters to editor, editorials, reviews)
Hand‐searched target journals	Actas Dermosifiliogr; Clin Cosmet Investig Dermatol; Dermatology; Dermatol Ther (Heidelb); J Am Acad Dermatol; J Eur Acad Dermatol Venereol

Extensive cross‐referencing and scanning of list of references of each potentially eligible study were performed, in order to minimize the risk of missing relevant investigations. Already available reviews were assessed for ensuring an adequate coverage but were not included in the present systematic review. No time or language filters were applied. The literature search was performed independently by two authors (Nicola Luigi Bragazzi, an expert in research methodology, and Giovanni Damiani, a dermatologist) and disagreements were solved by discussion until consensus was reached.

### 2.4 Data abstraction

The following data and information were extracted: surname of the first author, study year, country in which the study was performed, sample size, mean age, male percentage, skin phototype percentages, previous history of skin cancer and AK, previous treatment received, PROs/PROMs investigated and major findings and conclusions of the study. Two authors independently (Nicola Luigi Bragazzi and Giovanni Damiani) performed the data abstraction and any disagreement was solved through discussion until consensus was reached. Data abstraction was first pilot‐tested in a small sub‐set of studies randomly generated from sample of the included studies.

### 2.5 Literature synthesis

Data extracted was synthesized and displayed using tables, charts, and a narrative overview.

## 3 RESULTS

### 3.1 Literature search

**FIGURE 1:**
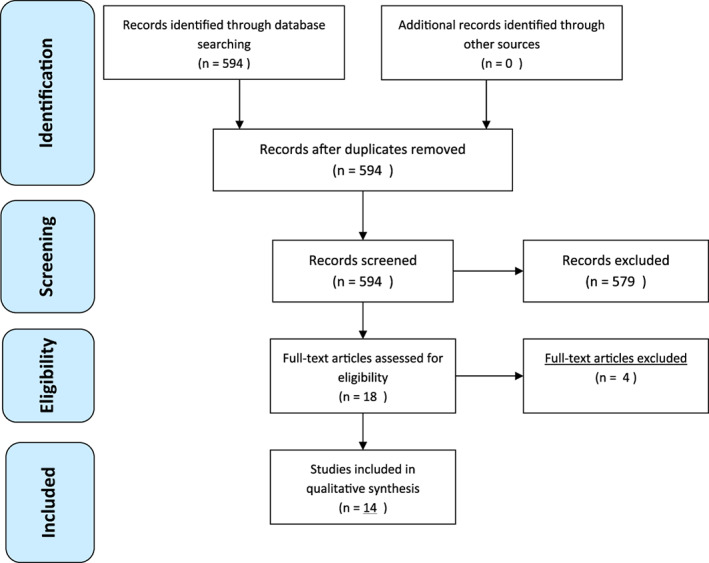
PRISMA flow diagram of the study

The initial literature search yielded a pool of 594 items (Figure 1). After screening title and/or abstract, 579 were removed. Three studies were excluded with reason (Table 2). Finally, 14 studies were included in the present systematic review (Table 3).

**TABLE 2:** Excluded studies list with the related reason

Excluded study with reason	Reason for exclusion
Kopasker et al^9^	Theoretical/methodological study (discrete experiment)
Longo and Serra‐Guillén^18^	Qualitative study (questionnaire‐based), without sufficiently detailed information on treatment
Salido‐Vallejo et al^7^	Qualitative study (focus group)
Philipp‐Dormston et al^11^	Indistinct PROs for single topical drug

**TABLE 3:** Included study list with their characteristics

First author	Study year	Country	Characteristics of the sample								
			Sample size	Age	Male	Skin phototypes	Ethnicity	History of skin cancer	Previous history of AK and treatment	Areas treated	Dimension of areas treated
Augustin et al.	2015	USA, Australia	1005 patients	65.1 y (34‐89 y)	74,70%	Majority with I and II phototypes	White/Caucasian (100%)	From 44.4% to 53.5%	Small percentage had been treated with imiquimod and topical fluorouracil (19.3% to 24.1%)	Face (43.8%), scalp (10.6%), arm (29.0%), back of hand (10.9%), chest (2.5%), leg (1.9%), back (0.7%), shoulder (0.6%)	NR
Berman et al.	2017	USA	188 patients from an initial list of 253 patients	From 64.0 y to 67.7	From 62.9% to 98.4% (depending on the group – based on the anatomical location depending on the group ‐ based on the anatomical location	I (from 7.9% to 19.4%), II (from 39.7% to 58.1%), III (from 19.4% to 47.6%), IV (from 3.2% to 4.8%)	From 98.4% to 100.0% (depending on the group – based on the anatomical location)	From 32.3% to 38.1% for NMSC	From 76.2% to 90.3%	Face/chest (33.5%), scalp (33.5%) and trunk or extremities (33.0%)	NR
Emilio et al.	2016	USA	28 patients, totaling 83 AK lesions	≥65 y	100%	NR	White/Caucasian (100%)	NR	NR	Face	25 cm^2^ contiguous
Gameiro et al.	2019	Brazil	37 patients, totaling 68 AK lesions	68.81 ± 7.72 y (49‐85 y)	51,35%	II (64.86%) and III (24.30%)	Caucasian (97.30%)	48,65%	54,05%	Nasal (33.82%), forehead (27.94%), malar (23.53%), scalp (14.70%)	91.18% (larger than 5 per 25 cm^2^ skin area)
Hanke et al.	2020	USA, Canada, Australia	729 patients	Ingenol mebutate group = 68.0 y (38‐91) and Vehicle group = 69.0 y(45‐91)	73,40%	I (18.9%), II (49.2), III (27.4%), IV (4.3%), V (0.1%)	Not Hispanic or Latino (99.6%), Hispanic or Latino (0.4%)	NR	NR	Face, Scalp and chest	Full scalp, full face, chest (250 cm2)
Jubert‐Esteve et al.	2015	Spain	19 patients	76.2 ± 7.7 y	89,50%	II (63.2%), III (21.1%), IV (15.8%)	NR	57.9% for NMSC	68.4%	Face (47.2%), scalp (42.1%), dorsum of hands (5.3%), forearm (5.3%)	97.0 ± 81.1 cm^2^
Neri et al.	2019	Italy	1136 patients	From 71.7 y to 73.8 y	73,20%	III (15.1%)	NR	NR	NR	Arm (4‐4.1%), trunk (4.7‐9.2%), face (23‐42.5%), scalp (47.3‐60.3%)	NR
Norrlid et al.	2018	Denmark, Sweden	446 patients	69.9 ± 9.0 y	56% (from 50%t o 73%, based on the pharmacological group)	I (17%), II (68%), III (14%), and 1 (IV)	NR	SCC (7%), BCC (35%), melanoma (5%)	75% (treated in 74% of patients)	Face (70%), scalp (22%), trunk (4%), extremities (4%)	23.8 ± 34.6 cm^2^
Platsidaki et al.	2020	Greece	440 patients	73.5 y (NR SD)	65,90%	I (7.3%), II (44.8%), III (39.3%), IV (8.0%), V (0.7%)	NR	NR	35.9% had previous AK treatment: prevalently with imiquimod, surgical/curettage and cry/liquid nitrogen	Unknown in 27.1%, and prevalently on face (36.7%) and scalp (16.1%)	NR
Schlaak et al.	2010	Germany	15 patients	>70	94%	NR	NR	NR	NR	Face/Head	NR
Segatto et al.	2013	Brazil	31 patients	74.4 ± 8.31(Diclofenac sodium) and 71.54 ± 8.60 (Fluorouracil)	46,40%	I (39.2%), II (50.0%), III (10.7%)	NR	15 (48.4) had a previous skin cancer not specified in the manuscript	NR	Face, Scalp and Back of the hands	NR
Stockfleth et al.	2017	Germany and UK	166 patients	72.2 ± 7.1 y	87,70%	I (8.6%), II (80.4%), III (10.4%), IV (0.6%)	White / Caucasian (100.0%)	NR	NR	Scalp (45.2%), Face/forehead (54.8%)	25 cm^2^
Strydom et al.	2018	New Zealand	75 patients out of an initial list of 100 patients	66 y (29‐88 y)	NR	NR	NR	NR	NR	Face	100 cm^2^
Waalboer‐Spuij et al.	2015	Netherlands	118 patients	67 ± 10 y	58%	NR	NR	Melanoma (5%), BCC (36%), SCC (11%), other skin malignancies	67%	Face/head/neck (66%), scalp (20%), torso (14%), arms (16%), legs (3%)	NR

**TABLE 3a:** 

First author	Study design	AK location	Treatment administered	Adherence to treatment	PROs	
					Instrument utilized	Main findings
Augustin et al.	Post‐hoc analyses from four phase‐III, multi‐center, randomized, double‐blind, vehicle‐controlled trials	Face, scalp, trunk and extremities	0.015% ingenol mebutate for 3 days or 0.05% for 2 days	From 98.2% to 98.7%	TSQM, Skindex‐16	Significant, positive associations between TSQM score and degree of *clearance were identified for patients in* Significant association between Skindex‐16 score and clearance for patients in the face/scalp group for change in symptoms. Emotions, and overall Skindex‐16 score from baseline.
Berman et al.	Phase II, multicenter, open‐label trial	Any part of the body (face, chest, scalp, trunk or extremities), with no lesions (from 74.2% to 85.7%), 1‐2 lesions (from 11.1% to 11.3%),	Ingenol disoxate gel applied once daily for 3 consecutive days (0.018% for face and chest, 0.037% for scalp and 0.1% for trunk or extremities)	97% (from 95% to 98%, depending on the specific area)	TSQM, cosmetic outcome questionnaire	Treatment satisfaction score ranging from 66.7/100 to 91.3/100. Based on the specific area, global satisfaction scores were 73.9/100, 79.7/100, 66.7/100 for the face/chest, scalp, and trunk/extremities groups, respectively
Emilio et al.	Prospective pilot study	Face	0.015% ingenol mebutate gel applied once daily for 3 consecutive days	NR	Skindex‐16	Mean overall scores improved from 24.5% at baseline to 15.5% as assessed on day 60. More in detail, treatment impacted on quality of life in a large and positive way for patients with mild and moderate LSR (Cohen's 2.1 and 1.8, respectively) and had, instead, little impact in patients with severe LSR (Cohen's d 0.2)
Gameiro et al.	Retrospective, descriptive, observational	Face and scalp	0.015% ingenol mebutate gel for 3 days	100%	*Ad hoc* non validated questionnaire	Perception of the treatment: great (75.68%)
						Discomfort: reasonable (40.54%), no discomfort (13.1%)
						Adverse reactions: erythema and local pruritus (16%) particularly disturbing
						Self‐esteem: improved in 97.30% cases
						Overall score: 9.4 (7–10)
Hanke et al.	Phase 3, randomized, parallel‐group, double‐blind, vehicle‐controlled trial	Face, scalp, chest	Ingenol mebutate 0.027% gel	4 patients drop‐out	TSQM and Skindex‐16	Patients who recieved ingenol mebutate were more satisfied.
Jubert‐Esteve et al.	Prospective, non randomized pilot study	Any part of the body	Imiquimod 5% and ingenol mebutate	0 drop out	TSQM and Skindex‐29	After treatment with ingenol mebutate, significant improvement was observed in the Skindex‐29 subscales relating to symptom severity, the patients' emotional state, and in the overall score. Imiquimod 5% and ingenol mebutate achieved higher median scores for effectiveness and global satisfaction than any other previous treatments (as measured by TSQM 1.4)
Neri et al.	Observational, multicentre, longitudinal, cohort study	Any part of the body	Ingenol mebutate, diclofenac, hyaluronic acid, Imiquimod 5%	46% avoided application within 2 h before bedtime, 14% washed the treated area erlier than 6 h post application. Patients undergoing long term treatment skip more than 20% of the applications in 5.2% of ingenol treated patients and in 74% of patients treated with different topical drugs	TSQM, PHQ4, *ad hoc* questionnaire‐based measures	Treatment satisfaction was higher for ingenol mebutate. Clarity in the communication between the physician and the patient was associated with a higher adherence and treatment satisfaction
Norrlid et al.	Observational, multicentre, real‐life study	Any part of the body, with 9.4 ± 8.2 lesions on average	diclofenac gel, imiquimod 3.75% or 5% or ingenol mebutate 150 μg/g or 500 μg/g	Treatment adherence was generally high, but higher for ingenol mebutate compared to both diclofenac (*p* < .001) and imiquimod (p = .007), possibly due to shorter treatment duration	TSQM‐9, MMAS, EQ‐5D‐5L, EQ‐VAS, AKQoL	Treatment satisfaction was higher for ingenol mebutate compared to patients treated with diclofenac
Platsidaki et al.	Noninterventional multicenter study	Face (61.6%), Scalp (32.5%) and others (5.9%)	Face and scalp: 150 mcg/g ingenol mebutate gel during 3 consecutive days; Other locations: 500 mcg/g ingenol mebutate gel for 2 days	100%	EQ‐5D questionnaire, EQ VAS and TSQM‐9	Patients reported high satisfaction, especially in case of complete AK clearance.
Schlaak et al.	Single center, prospective study	Face/Head	Solution of 5 mg fluorouracil (0.5%) and 100 mg salicylic acid (10%) 3 times per day for 4 weeks	1 patient drop‐out due to side effects	Treatment satisfaction VAS	Patients satifaction was “good”
Segatto et al.	Randomized, parallel‐group clinical trial	Face, Scalp and Back of the hands	3% diclofenac sodium with 2.5% hyaluronic acid gel twice daily for 12 weeks vs. 5% 5‐Fluorouracil cream twice daily for 4 weeks	3 patients drop‐out in the 5FU group.	Treatment satisfaction VAS	In relation to satisfaction regarding the adverse effects, the group treated with DFS showed higher satisfaction compared to the group treated with 5‐FU, with 93.3% and 38.4% of highly satisfied patients, respectively. Regarding the patients' evaluation, most were highly satisfied with the improvement of the lesions in both groups, with no statistically significant difference. When considering the degree of improvement, more than half of the patients (54%) in the group treated with 5‐FU considered themselves to be fully healed, compared to 20% in the group treated with DFS.
Stockfleth et al.	Phase III, multicenter, randomized, double‐blind, vehicle‐controlled study	Scalp and face/forehead	5‐FU 0.5% plus salicylic acid 10%	2 patients drop‐out	TSQM and DLQI	Treatment satisfaction scores were higher in treated patients when compared to vehicle. No statistically significant differences were observed between the study arms for the TSQM convenience and side effect domain scores.
Strydom et al.	Single‐center, prospective, questionnaire‐based study	Face	0.015% ingenol mebutate applied once daily for 3 consecutive days, over areas up to 100 cm^2^	NR	*Ad hoc*, nonvalidated 11‐item questionnaire	Treatment satisfaction was rather high (86‐89%). Pretreatment education was appreciated by all patients.
						58% patients experienced moderate‐to‐severe pain. 51%, 41% and 9% found their appearance, pain and anxiety particularly distressing, respectively. 31% would have discontinued the treatment in case of self‐application, with 82% preferring in‐clinic application
Waalboer‐Spuij et al.	Multicenter open‐label study	Any part of the body, with 1 lesion (25%), 2–4 lesions (13%), 5‐9 lesions (29%), ≥10 lesions (31%)	5% imiquimod cream once daily, 3 days per week, for 4 weeks	6‐7% of patients decided to discontinue the therapy	Skindex‐17, TSQM, SCI adapted to AK	Imiquimod had no impact on health‐related quality of life. Overall treatment satisfaction was less than 60/100

Abbreviation: NR ‐ Not reported.

### 3.2 Characteristics of the included studies

Included studies were published between 2010 and 2020. Only four studies were clinical trials.^10^, ^23^, ^24^, ^25^ Investigations were designed prevalently as national studies (10/14)^6^, ^8^, ^10^, ^12^, ^15^, ^16^, ^17^, ^22^, ^23^, ^26^ and carried out in North America,^10^, ^15^ Europe,^8^, ^16^, ^17^, ^22^, ^26^ South America,^6^, ^23^ and Australia.^12^ Brazil was the only represented South American country,^6^, ^23^ and no data were present to represent Asian and African countries. Sample size ranged from 15^22^ to 1136^8^ patients, totaling a sample of 4433 patients for all included studies.^6^, ^8^, ^10^, ^12^, ^13^, ^15^, ^16^, ^17^, ^19^, ^22^, ^23^, ^24^, ^25^, ^26^ The examined population were prevalently Caucasian males with face and scalp AK.

AK lesions involving any part of the body were assessed by most of the studies,^8^, ^10^, ^13^, ^16^, ^17^, ^23^, ^25^, ^26^ whereas four studies focused on only face and/or scalp treatment.^6^, ^22^, ^24^ 5% to 57.9% of patients had a previous history of skin cancer, while 19.3% to 90.3% had been previously treated for AK.^6^, ^8^, ^10^, ^12^, ^13^, ^15^, ^16^, ^17^, ^19^, ^22^, ^23^, ^24^, ^25^, ^26^ The majority of the patients belonged to Fitzaptrick's skin phototypes I and II.^6^, ^8^, ^10^, ^12^, ^13^, ^15^, ^16^, ^17^, ^19^, ^22^, ^23^, ^24^, ^25^, ^26^

### 3.3 Utilization of PRO instruments

The included studies reported several PRO instruments that could be categorized into AK‐specific and non‐specific instruments. Only one study utilized a validated AK‐specific instrument, the Actinic Keratosis Quality of Life questionnaire (AKQoL),^13^ that assesses quality of life but not patients' satisfaction with treatment.^27^

Non AK‐specific validated instruments reported include Treatment Satisfaction Questionnaire for Medication (TSQM),^8^, ^10^, ^13^, ^16^, ^17^, ^19^, ^24^, ^25^, ^26^ Skindex‐16,^15^, ^19^, ^25^ Skindex‐17,^17^ Skin Cancer Index (SCI),^17^ Patient Health Questionnaire‐4 (PHQ‐4)^8^, EuroQoL 5‐level EQ‐5D version (EQ‐5D‐5L),^13^, ^26^ EuroQol Visual Analogue Scale (EQ‐VAS),^13^, ^26^ and Dermatology Life Quality Index (DLQI).^24^

Four studies utilized ad‐hoc, non‐validated questionnaires.^6^, ^10^, ^12^, ^23^ Five studies utilized only one PRO (ad‐hoc questionnaire^6^, ^12^, ^23^ vs validated questionnaire^11^, ^15^), while 11 studies used more than one PRO instrument (only validated questionnaires^13^, ^17^, ^19^, ^24^, ^25^, ^26^ vs validated and not validated questionnaires^8^, ^10^). One study used a validated questionnaire, the Morisky Medication Adherence Scale (MMAS), to quantify patients' adherence.^13^

### 3.4 Patient‐reported outcomes

Ten studies evaluated a single AK topical drug (imiquimod 5%,^17^ ingenol mebutate,^6^, ^12^, ^15^, ^19^, ^25^, ^26^ ingenol disoxate,^10^ 5‐FU^22^, ^24^) finding acceptable overall satisfaction. Four studies compared different topical treatments (diclofenac vs 5‐FU,^23^ diclofenac vs imiquimod vs ingenol mebutate,^8^, ^13^ ingenol mebutate vs imiquimod 5%^16^).

Ingenol mebutate and ingenol disoxate appeared to be well‐tolerated by patients, with rather high treatment satisfaction scores and improved quality of life.^6^, ^8^, ^10^, ^12^, ^13^, ^15^, ^16^, ^17^, ^18^, ^19^, ^25^, ^26^ In a small pilot study (n=19), both imiquimod 5% and ingenol mebutate achieved higher median scores for effectiveness and global satisfaction than any other previous treatments. However, ingenol mebutate achived higher median score on convenience. ^16^ Ingenol mebutate was superior to diclofenac in terms of satisfaction and treatment adherence.^8^, ^13^ Diclofenac caused fewer adverse events (erythema, edema, crusts and itching) than 5‐FU.^23^

When compared with other available pharmacological options, ingenol mebutate, a topical treatment with simpler and shorter‐duration regimen, appeared to be superior to the comparators from the patients' perspective.

Adherence to the treatment was generally very high for shorter‐duration treatments, with few patients reporting treatment discontinuation due to side effects.^6^, ^8^, ^10^, ^12^, ^13^, ^15^, ^16^, ^17^, ^19^, ^22^, ^23^, ^24^, ^25^, ^26^

No data was identified for piroxicam.

## 4 DISCUSSION

Actinic keratoses are chronic, recurring lesions and represent a substantial disease burden due to their high prevalence and associated risk of frank malignancy. Furthermore, AKs are quite distressing to many patients not only as a cosmetic liability, but also treatment‐related local skin reactions occurring on habitually exposed body locations, particularly the face and bald scalp, with significant impact on daily living activities such as work and social life.^1^

This systematic review explored PROs/PROMs regarding topical field‐directed treatment for AK. Investigating patients' perspective is crucial when different treatment options exist, especially when facial and scalp lesions are detrimental in terms of perceived quality of life. Most included studies employed a small sample size, with few being clinical trials and only one utilized a validated instrument specifically developed for AK. Development and use of psychometrically sound and validated instruments may advance the field.

One important limiting factor for some AK topical treatments is poor tolerability due to local skin reaction. A prospective, open‐label, multicenter study by Stough et al included 277 patients treated with once daily application of 5‐FU 0.5% cream for up to 4 weeks. In an interim analysis of the face and scalp findings, severe local skin reactions (LSRs) developed in nearly 20% of patients.^28^ Severe LSRs such redness, pain, erosions and ulcerations can have an impact on patients' social activities and hence poor adherence to therapy.^29^

Imiquimod showed efficacy^17^ but lower tolerability and patient's satisfaction than ingenol mebutate.^8^, ^13^, ^30^ Furthermore, imiquimod can trigger latent, unpredictable inflammatory dermatoses, particularly on the scalp^31^ and also may trigger distant inflammatory mucosal reactions.^30^

In addition to tolerability, prolonged treatment duration is a significant factor contributing to nonadherence and nonpersistence to topical treatments. In a community‐based, cross‐sectional study, patient‐applied topical therapies that required less frequent application and have shorter treatment duration were associated with better adherence rates.^32^ Our study results shows that patient satisfaction corresponds with shorter duration of topical treatment.

Involving patients and empowering them, implementing patient‐centered care and taking into account patients' preferences and perspectives could enhance and improve their health status, paving the way also for personalized management options. Within this conceptual framework, patient education is fundamental, especially for multiple AK lesions. Pretreatment education is appreciated by patients and results in a high treatment satisfaction scores (in the range 86‐89%).^12^

New venues in the field could include the study of the feasibility of exploiting educational videos or the new information and communication technologies (ICTs) to improve patients' satisfaction and adherence to the treatment.^14^

On the other hand, despite its transparency, rigor, methodological strengths, and reproducibility, the present study is not without limitations. The major shortcomings are given by the small studies included and by mining only PubMed/Central. The high heterogeneity among studies did not enable to carry out a meta‐analysis.

## 5 CONCLUSIONS

Actinic keratoses are precursors for invasive cutaneous squamous cell carcinoma. Different treatment options exist for actinic keratoses. Topical therapies with simpler and shorter‐treatment regimen appeared to achieve high patient satisfaction, better adherence and improve overall quality of life. Incorporating patients' perspective in clinical trials may be helpful. Well‐designed studies utilizing validated PRO instuments should help define patients' preferences.

## CONFLICT OF INTEREST

Ayman Grada, MD, MS is an employee of Almirall LLC. Steve Feldman, MD, PhD received research, speaking and/or consulting support from Galderma, GSK/Stiefel, Almirall, Alvotech, Leo Pharma, BMS, Boehringer Ingelheim, Mylan, Celgene, Pfizer, Ortho Dermatology, Abbvie, Samsung, Janssen, Lilly, Menlo, Helsinn, Arena, Forte, Merck, Novartis, Regeneron, Sanofi, Novan, Qurient, National Biological Corporation, Caremark, Advance Medical, Sun Pharma, Suncare Research, Informa, UpToDate and National Psoriasis Foundation. He consults for others through Guidepoint Global, Gerson Lehrman and other consulting organizations. He is founder and majority owner of www.DrScore.com. He is also a founder and part owner of Causa Research, a company dedicated to enhancing patients' adherence to treatment.

## AUTHOR CONTRIBUTIONS

CRediT (Contributor Roles Taxonomy): Conceptualization: Ayman Grada, Steven R. Feldman, Nicola Luigi Bragazzi, and Giovanni Damiani; Methodology: Giovanni Damiani and Nicola Luigi Bragazzi; Software: Nicola Luigi Bragazzi; Validation: Ayman Grada, Nicola Luigi Bragazzi, and Giovanni Damiani; Formal analysis: Nicola Luigi Bragazzi; Investigation: Ayman Grada, Steven R. Feldman, Nicola Luigi Bragazzi, and Giovanni Damiani; Resources: Ayman Grada; Data curation: Nicola Luigi Bragazzi and Giovanni Damiani; Writing: Ayman Grada, Nicola Luigi Bragazzi, Giovanni Damiani; Revision & Editing: Ayman Grada, Steven R. Feldman, Nicola Luigi Bragazzi, and Giovanni Damiani; Visualization: Giovanni Damiani and Nicola Luigi Bragazzi; Supervision: Giovanni Damiani, Ayman Grada; Project Administration: Nicola Luigi Bragazzi, and Giovanni Damiani. All authors read and approved the final version of the manuscript.

## Data Availability

Data sharing is not applicable to this article as no new data were created or analyzed in this study.
